# Evaluation of Prevalence of the Sarcopenia Level Using Machine Learning Techniques: Case Study in Tijuana Baja California, Mexico

**DOI:** 10.3390/ijerph17061917

**Published:** 2020-03-15

**Authors:** Cristián Castillo-Olea, Begonya Garcia-Zapirain Soto, Clemente Zuñiga

**Affiliations:** 1 eVIDA Research Group, University of Deusto, Bilao 48007, Spain; mbgarciazapi@deusto.es; 2 Geriatric, General Hospital of Tijuana, Tijuana 22195, Mexico; zclemente@hotmail.com

**Keywords:** machine learning, sarcopenia level, prevalence

## Abstract

The article presents a study based on timeline data analysis of the level of sarcopenia in older patients in Baja California, Mexico. Information was examined at the beginning of the study (first event), three months later (second event), and six months later (third event). Sarcopenia is defined as the loss of muscle mass quality and strength. The study was conducted with 166 patients. A total of 65% were women and 35% were men. The mean age of the enrolled patients was 77.24 years. The research included 99 variables that consider medical history, pharmacology, psychological tests, comorbidity (Charlson), functional capacity (Barthel and Lawton), undernourishment (mini nutritional assessment (MNA) validated test), as well as biochemical and socio-demographic data. Our aim was to evaluate the prevalence of the level of sarcopenia in a population of chronically ill patients assessed at the Tijuana General Hospital. We used machine learning techniques to assess and identify the determining variables to focus on the patients’ evolution. The following classifiers were used: Support Vector Machines, Linear Support Vector Machines, Radial Basis Function, Gaussian process, Decision Tree, Random Forest, multilayer perceptron, AdaBoost, Gaussian Naive Bayes, and Quadratic Discriminant Analysis. In order of importance, we found that the following variables determine the level of sarcopenia: Age, Systolic arterial hypertension, mini nutritional assessment (MNA), Number of chronic diseases, and Sodium. They are therefore considered relevant in the decision-making process of choosing treatment or prevention. Analysis of the relationship between the presence of the variables and the classifiers used to measure sarcopenia revealed that the Decision Tree classifier, with the Age, Systolic arterial hypertension, MNA, Number of chronic diseases, and Sodium variables, showed a precision of 0.864, accuracy of 0.831, and an F1 score of 0.900 in the first and second events. Precision of 0.867, accuracy of 0.825, and an F1 score of 0.867 were obtained in event three with the same variables. We can therefore conclude that the Decision Tree classifier yields the best results for the assessment of the determining variables and suggests that the study population’s sarcopenia did not change from moderate to severe.

## 1. Introduction

Sarcopenia is a disease of multifactorial origin. The main factors are malnutrition, neuromuscular, and mitochondrial dysfunction as well as hormonal changes. The disease leads to a loss of muscle mass in older adults. In Mexico, there are almost 12 million people who suffer from sarcopenia and do not know it, with a prevalence of 48.5% in women and 27.4% in men [1]. Around 50 years of age, muscle mass decreases from 1% to 2% per year, and muscle strength has an annual decrease of 1.5% between 50 and 60 years of age, and then 3% every year after. Between 5% and 13% of people between 60 and 70 years of age and 11% to 50% of people 80 years of age or older have sarcopenia. Several studies have reported that hospitalization reduces muscular mass and strength in elderly patients [2,3]. 

As Machine Learning (ML) is a technique consisting of a dataset that identifies relationships between features and algorithm outputs, it was applied in this study. By using algorithms, it is possible to develop techniques that allow the computer to “learn” to classify features, creating algorithms capable of generalizing data from unstructured information as samples [4,5]. Machine learning is very useful in the early diagnosis of affliction and diseases. The algorithms that incorporate it can “learn” when the conditions exist for a patient to suffer from a disease. If the algorithm detects characteristics in the patient that can lead to a disease, it will warn the patient of it.

Supervised learning in ML is a set of techniques that allow for making predictions based on behavior or features analyzed from known data (input). A supervised learning algorithm analyzes the input data (characteristics analyzed) and produces an output which is the variable being predicted [6,7]. By selecting the features, relationships and patterns can be established between the data and features about which we wish to make a prediction [5,8].

A wide variety of algorithms are used in Machine Learning, some of which are extremely popular like Nearest Neighbors, Linear Support Vector Machines (SVM), Radial Basis Function (RBF) SVM, Gaussian process RBF, Decision Tree, Random Forest, AdaBoost, Gaussian Naive Bayes, etc. However, there is no predefined validated model that works efficiently and effectively on all databases. One or several algorithms must be selected to create a model and later validate it to ensure optimal functions, according to the type of data and the output variable being predicted [9,10]. 

The objective of this article is to classify the variables that determine the level of moderate or severe sarcopenia, depending on the case, with the use of machine learning algorithms, which will allow us to know the prevalence of sarcopenia and the status of chronic patients at the Hospital General de Tijuana. The patients that attend the hospital have only scarce resources.

## 2. Materials and Methods

This article is based on a study of the level of sarcopenia in older patients at the Tijuana General Hospital, specifically from the geriatrics section. In 2017, the older adult population in Tijuana was 85,259, of which 65% were attended at the Hospital [11]. This public hospital attends a population with limited economic resources. The descriptive observational study evaluated a group of patients diagnosed with moderate or severe sarcopenia. 

### 2.1. Sample Size

The necessary sample size was estimated by taking into account the expected prevalence in studies made via bioimpedance analysis in older adults, 17% [12], assuming a 5% margin of error and 95% confidence interval. According to these criteria, a total of 166 patients were needed to obtain the desired results. 

The patients were from the following areas of Baja California: Tijuana, Ensenada, Tecate, Mexicali, and Rosarito. Patients who had been diagnosed with moderate to severe sarcopenia and who were willing to take part in the study signed an informed consent. No physically or psychologically dependent patients were included in the study. Older adults are sedentary, that is, they do not perform any physical activity. The diagnosis of sarcopenia was made by geriatricians at the Tijuana General Hospital. The research included patients’ medical history, pharmacology, psychological tests, comorbidity (Charlson), functional capacity (Barthel and Lawton), malnutrition (mini nutritional assessment (MNA) validated test), as well as biochemical and socio-demographic data. 

### 2.2. Database

**Figure 1:**
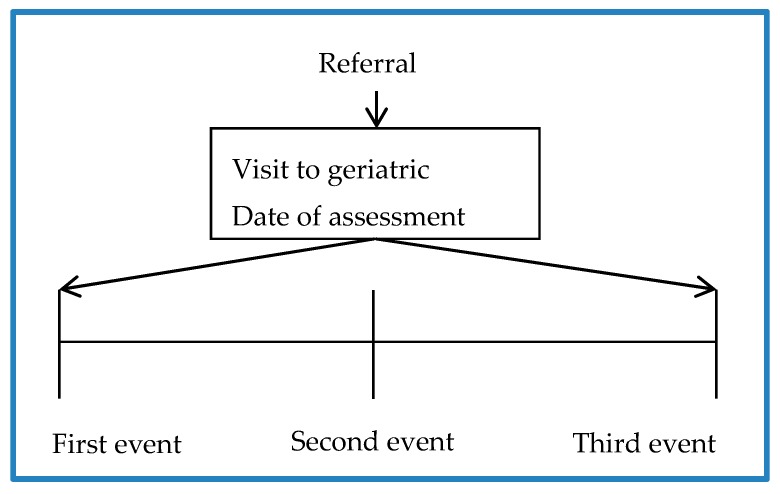
Study timelines.

Information on 166 patients with 99 variables for each one was compiled to create the database. The study timeline used to obtain information was at the beginning of the study (first event), 3 months later (second event), and 6 months later (third event) (see Figure 1). 

Table 1 shows the criteria applied by gender to assess the patients at Tijuana General Hospital. This hospital attends a population with limited economic resources from areas of Baja California, specifically Tijuana, Ensenada, Tecate, Mexicali, and Rosarito. As can be observed in Table 1, there is a higher percentage of women than men.

### 2.3. Machine Learning Models

According to clinical practice manuals like ResOhma, variables which provide post diagnostic information were eliminated to create machine learning models [13,14,15]. During the process, the following 10 models were used: Nearest Neighbors [3], Linear SVM (Support Vector Machines), RBF SVM (Radial Basis Function), Gaussian process, Decision Tree, Random Forest, MPL, AdaBoost, Gaussian Naive Bayes, and QDA (Quadratic Discriminant Analysis) [4,16]. A ranking was elaborated to extract the variables having the greatest impact on the quality of the different models created. The ranking classifies the variables by assigning a value to each one, with the lowest value indicating the highest impact. 

#### Model Classification

The dataset for the first event was initially split into 90% for the training group and 10% for the test group, preserving the distribution of the classes of elements. Taking the size of the dataset into account, a stratified 5-fold cross-validation that preserves the balance of classes in the different folds was used instead of creating a validation group from the training group.

Each dataset was evaluated on different Machine Learning models for classification, evaluating the following metrics for each one: Accuracy, F1, and Precision (see Table 2). 

Once the characteristics were selected, the training was performed and the 10 models for each of the datasets were cross-validated at each information collection event. In other words, at the first, second, and third events they were examined to determine if they knew the patients’ evolution. 

## 3. Results

### 3.1. Significant Variables

Table 3 shows the variables used in each dataset that was created. Each dataset includes a number of variables in descending order of importance. 

Table 3 shows that the Age variable had the greatest impact. In the first, second, and third events, we observe that the variables have the same order of importance.

Table 4 shows the classifiers that obtained the best results in the first event in relation to dataSET 1, dataSET 2, dataSET 3, and dataSET 4.

Table 5 shows the classifiers that obtained the best results in the second event in relation to dataSET 1, dataSET 2, dataSET 3, and dataSET 4. 

In general, it can be seen that the data were equal or even better results were obtained for dataSET 1 (which contains the four variables which were considered most important) than when taking variables which were listed as less important in the ranking elaborated. This refers to the first and second event. The reason for this is that the models trained on a lower number of variables are of good quality and are not exposed to overtraining due to an excess of information.

The RBF SVM classifier obtained good results for all the metrics, regardless of the dataset used in both the first and second event. The Decision Tree classifier obtained better results using dataSET 1 and dataSET 3 in the first event. However, the best results were obtained using dataSET 1 in the second event. DataSETs 1, 2, and 4 obtained the best results in the first event with the Random Forest classifier. However, better results were obtained with 2 and 4 in the second event. The Linear SVM classifier obtained better results for dataSETs 2 and 3 and, on the other hand, it did not give optimum results in the second event. The Quadratic Discriminant Classifier (QDA) obtained better results for dataSET 1.

Table 6 shows the patients’ results obtained in the third event. It indicates that the Decision Tree classifier obtained an accuracy of 0.830, precision of 8.64, and an F1 score of 0.900 with dataSET 1. This is the best classifier for dataSET 1.

Table 7 shows that the RBF SVM classifier obtained better results for dataSET 2, dataSET 3, and dataSET 4, yielding an accuracy of 0.813, precision of 0.813, and an F1 score of 0.897 for the three dataSETs. The Random Forest classifier showed an accuracy of 0.813, precision of 0.817, and an F1 score of 0.896 for dataSET 3. It obtained an accuracy of 0.813, precision of 0.813, and an F1 score of 0.897 F1 for dataSET 4. The Gaussian process classifier obtained an accuracy of 0.813 accuracy, precision of 8.13, and an F1 score of 0.897. 

### 3.2. Final Results 

Table 7 indicates that, in general, the Decision Tree classifier obtained better results for dataSET 1 in the three events (see Table 7). 

**Figure 2:**
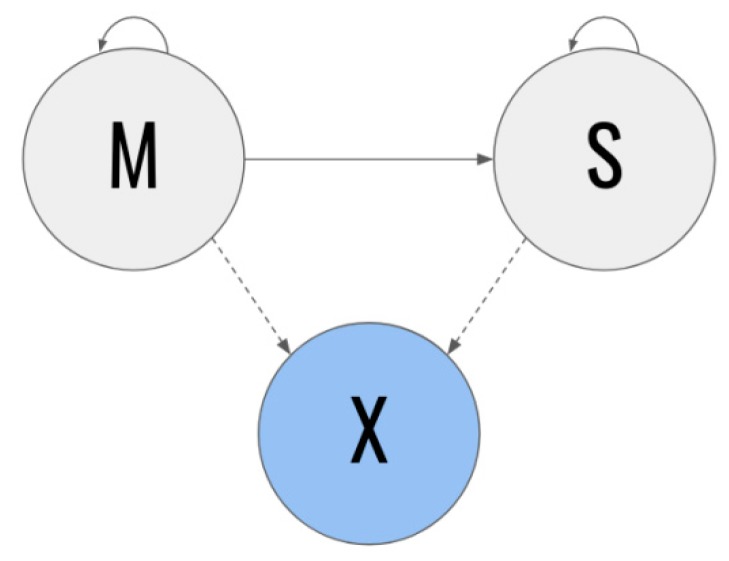
Model based on Markov hidden chains.

Due to the above, we can therefore suggest that using the Age, Systolic arterial hypertension, MNA, Number of chronic diseases, and Sodium variables to train the Decision Tree classifier helps in assessing patients diagnosed with sarcopenia. In order to model and assess the dynamics over time for a patient with the determining variables in dataSET 1, a model based on a hidden Markov model was developed. The model is shown in Figure 2 [17].

In the Markov chains-based model, the gray states represent the unobservable states and the blue states represent the observable states. In addition, the solid lines represent transition probabilities over time and the dotted lines represent emission probabilities. The **M** state specifically represents the patients with a diagnosis of moderate sarcopenia at time *t*, whereas the **S** state represents the patients diagnosed with severe sarcopenia at said time *t*. A patient in the **M** state at a certain time *t* (in other words, who has moderate sarcopenia at that time) has some probability of the condition worsening to state **S** at the following point in time *t* + 1. On the other hand, they also have a certain probability of remaining in state **M**. However, patients with severe sarcopenia do not have the possibility of returning to state **M** at some time in the future [18,19].

Due to patients being in state **M** or **S** at time period *t,* and it is not possible to directly infer their current condition, it is possible to observe the **X** vector (characteristic vector) instead. For this section, we chose to include the characteristics with the greatest weight on the classifiers in **X**. 

A Markov hidden model may formally represent the five-tuple (Q, π, A, B), where:

- Q: the set of states, Q = {s1, s2}, where s1 = M and s2 = S;
- **A** = {aij}: state transition matrix
  o aij = P (qt + 1 = sj | qt = si);
- **B** = {bj(k)}: State emission probability j
  o bj(k) = P (k en t | qt = sj);
- π = {πi}: initial distribution
  o πi = P (q0 = si).

Due to an observation sequence O = {o1, o2,.., oT} and a model λ = {Q, A, B, π}, it is possible to compute the probability that said sequence comes from the model raised. In other words, P(O | λ), using the advanced regression algorithm. Taking this point into account, it is possible to generate an observation sequence for each classifier and use a Markov hidden model to verify whether it aligns with our priorities.

The distribution of the determining variables by the Decision Tree classifier is as follows: 

#### 3.2.1. Sodium

**Figure 3:**
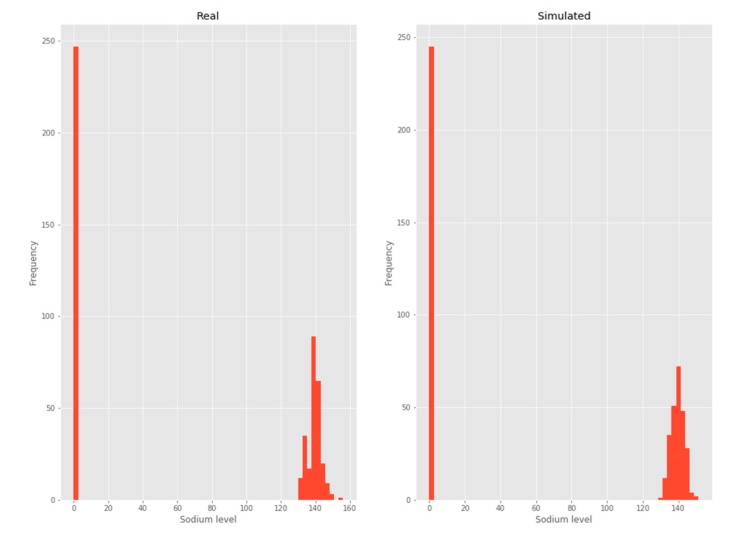
Sodium.

The Sodium variable was modeled as a mix between a normal distribution and dirac delta. In this case, the parameters to be estimated are those that correspond to the normal distribution and those that correspond to the "vanishing” values. A frequency histogram is shown below for the best fit curve of the previously described characteristics over the complete dataset (Figure 3).

#### 3.2.2. Number of chronic diseases

**Figure 4:**
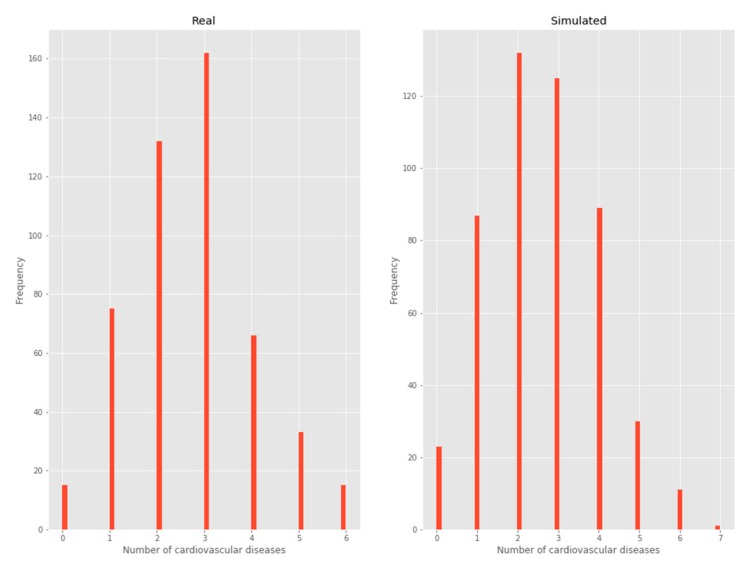
Number of chronic diseases.

By considering each cardiovascular disease as a Bernoulli trial [12], the binomial distribution to model each patient’s number of cardiovascular diseases emerges naturally, as shown below in the frequency histogram (Figure 4).

#### 3.2.3. Systolic arterial hypertension

**Figure 5:**
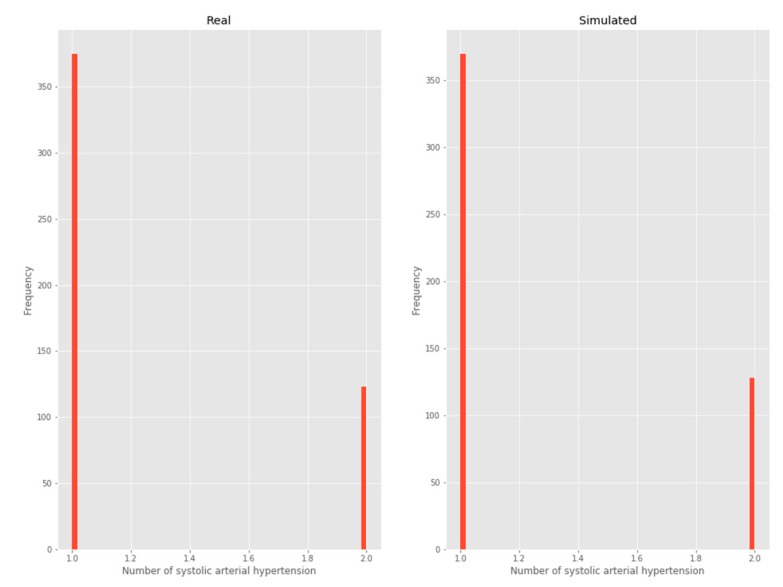
Systolic arterial hypertension.

As this is a variable with two possible outputs, it was considered a Bernoulli distribution (Figure 5).

#### 3.2.4. Age

**Figure 6:**
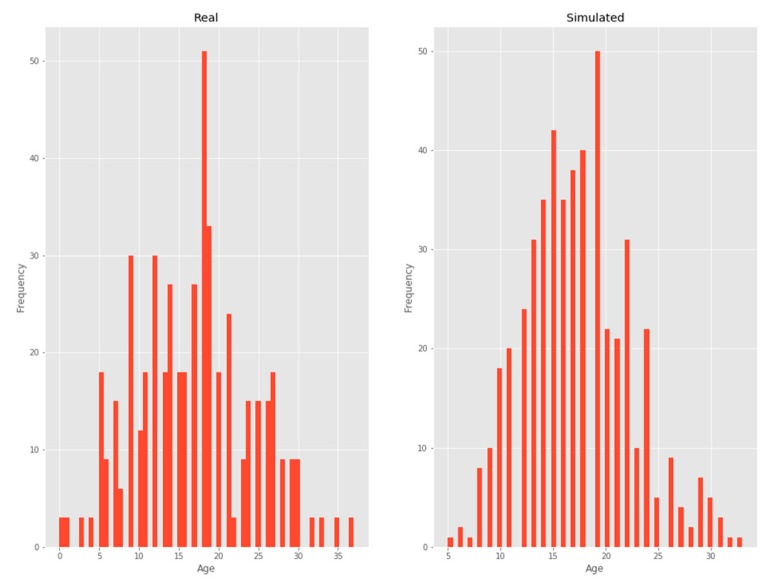
Age.

A negative binomial distribution was chosen as it was the one that minimized KL divergence (Figure 6).

#### 3.2.5. MNA

**Figure 7:**
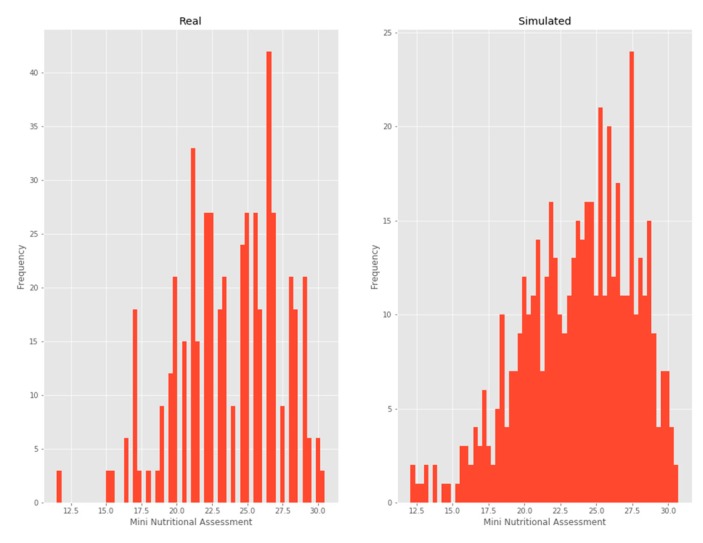
Mini-nutritional assessment.

A beta distribution was chosen as it was the one that minimized the KL divergence (Figure 7).

When analyzing different paths on the model, it was observed that the sequences that contained state changes between times were especially penalized by the model raised. Further analysis of the chain clearly showed the reason: the transition probability from the **M** to **S** state is always null. This is due to the fact that no patient’s condition became worse during the time recorded. It is therefore not possible to assign adequate priors to the phenomenon.

## 4. Discussion

There is research that studies the CT scan using automatic learning techniques, which evaluate the muscle volume of adults with sarcopenia, and the results are between 0.80 and 0.87 of precision [20,21,22,23,24]. A study on sarcopenia with similar technical characteristics to our study [20] used four classifiers, Random Forest, SVM, Gradient Boosting, and Logistic regression, with Random Forest as the best classifier, obtained an accuracy of 0.82. The article for the measurement of variables involved in the development of sarcopenia, using forecasting networks based on automatic learning approaches, where the results show an accuracy of 82%, analyzed 114 variables in this study [25].

In our study on the diagnostic detection of sarcopenia over a six-month period, 99 variables from 166 patients were analyzed, obtaining an accuracy of 0.825, precision of 0.867, and an F1 score of 0.895, which is the harmonic mean of accuracy and recovery, using the Decision Tree Classifier. This compared with the study of [26] makes a difference in the dataset of 4020 patients with a conclusion of accuracy between 0.78 and 0.82. Patients evolved favorably since there were no changes of moderate to severe sarcopenia during the study, although four patients died due to external causes. A limited version of this study was conducted for six months, during which four people died from external causes. In [27], the duration of the study was 12 months but the results showed an accuracy of 0.82.

## 5. Conclusions

Our study suggests that the Age, Systolic blood pressure, MNA, Number of chronic diseases, and Sodium variables are determinants when evaluating patients with moderate and severe sarcopenia. Therefore, these variables can complement the assessment of sarcopenia based on ResOhms, MMI, hand grip strength, calf circumference, and walking speed, which are the standard variables for the assessment of this pathology.

A limitation of this study is that it was conducted over one year ago. This stage of the project presents results from data obtained in nine months. As for future lines of research, data are being collected for the last period of the study to complete the follow-up year. The aim is to generate a predictive model to prevent the deterioration of the patient diagnosed with sarcopenia, that is to say, to know which variables are decisive for a patient to remain at a moderate or severe level, or to change from severe to moderate.

## Figures and Tables

**Table 1:** Assessment criteria at Tijuana General Hospital [11].

	Gender	MMI	Hand Grip Strength	Walking Speed
Women	65%	<6.1 kg/m^2^	<20	<0.8
Men	35%	<8.5 kg/m^2^	<30	<0.8

**Table 2:** Metrics.

Metric	Formula
Accuracy	$Acc=\frac{\left(TP+TN\right)}{\left(TP+TN+FP+FN\right)}$
Precision	$Prec=\frac{TP}{\left(TP+FP\right)}$
F1	$F1=2\times \frac{P\cdot R}{P+R}$

**Table 3:** DataSET group.

**DataSET 1**
Age, Systolic arterial hypertension, mini nutritional assessment (MNA), Number of chronic diseases, Sodium
**DataSET 2**
Age, Systolic arterial hypertension, MNA, Number of chronic diseases, Sodium, Drugs, Lawton
**DataSET 3**
Age, Systolic arterial hypertension, MNA, Number of chronic diseases, Sodium, Drugs, Lawton, Hb, Major neurocognitive disorder, Dementia, Occupation, Means of support
**DataSET 4**
Status, Gender, Age, Level of education, Literacy, Civil status, Carer, Religion, Residence, Occupation, Economy, Means of support, Eyesight, Visual aid, Hearing, Hearing aid, Number of chronic diseases, Systolic arterial hypertension, Major neurocognitive disorder, PARKIN, HIPOT, HIPERT’, CANCER, COPD, Dyslipidemia, Chronic renal insufficiency, Other, Hepatic insufficiency, Smoking, Alcoholism, Drug use, Biomass exposure, MMSE, GDS, Depression, Barthel, Falls, Number of falls, Ulcers, Norton, Lawton, MNA, Charlson, Height in mts, Dementia, Cognition, Cerebrovascular disease, Infection, Pain, Cancer, Hb, Urea, Creatinine, Albumin, Glucose, ’Sodium’

**Table 4:** Comparison of results.

CLASSIFIERS	DataSET 1			DataSET 2			DataSET 3			DataSET 4			DataSET
	ACC	F1	P	ACC	F1	P	ACC	F1	P	ACC	F1	P	Final
RBF SVM	**0** **.** **825**	**0** **.** **902**	**0** **.** **828**	**0** **.** **813**	**0** **.** **897**	**0** **.** **813**	**0** **.** **813**	**0** **.** **897**	**0** **.** **813**	**0** **.** **813**	**0** **.** **897**	**0** **.** **813**	**1, 2, 3, 4**
Decision Tree	**0** **.** **831**	**0** **.** **900**	**0** **.** **864**	0.795	0.879	0.844	**0** **.** **819**	**0** **.** **897**	**0** **.** **84**	0.765	0.842	0.866	**1, 3**
Random Forest	**0** **.** **825**	**0** **.** **901**	**0** **.** **836**	**0** **.** **825**	**0** **.** **902**	**0** **.** **827**	0.795	0.886	0.810	**0** **.** **801**	**0** **.** **89**	**0** **.** **811**	**1, 2, 4**
Linear SVM	0.813	0.897	0.813	**0** **.** **813**	**0** **.** **897**	**0** **.** **813**	0.813	0.897	0.813	**0** **.** **765**	**0** **.** **842**	**0** **.** **866**	**2, 3**

ACC = Accuracy, P = Precision. Bold: Better results

**Table 5:** Comparison of results.

CLASSIFIERS	DataSET 1			DataSET 2			DataSET 3			DataSET 4			DataSET
	ACC	F1	P	ACC	F1	P	ACC	F1	P	ACC	F1	P	Final
RBF SVM	**0.81**	**0.896**	**0.817**	**0.813**	**0.897**	**0.81**	**0.81**	**0.9**	**0.81**	**0.81**	**0.9**	**0.81**	**1, 2, 3, 4**
Decision Tree	**0.831**	**0.900**	**0.864**	0.807	0.885	0.855	0.783	0.870	0.837	0.662	0.704	0.873	**1**
Random Forest	0.807	0.891	0.819	**0.819**	**0.899**	**0.82**	0.795	0.885	0.813	**0.81**	**0.89**	**0.81**	**2, 4**
MPL	**0.84**	**0.905**	**0.855**	0.783	0.870	0.853	0.747	0.844	0.839	0.716	0.817	0.842	**1**

ACC = Accuracy, P = Precision. Bold: Better results

**Table 6:** Comparison of results.

CLASSIFIERS	DataSET 1			DataSET 2			DataSET 3			DataSET 4			DataSET
	ACC	F1	P	ACC	F1	P	ACC	F1	P	ACC	F1	P	Final
RBF SVM	0.807	0.893	0.812	**0.813**	**0.897**	**0.813**	**0.813**	**0.897**	**0.813**	**0.813**	**0.897**	**0.813**	**2, 3, 4**
Decision Tree	**0.825**	**0.895**	**0.867**	0.789	0.872	0.852	0.753	0.848	0.825	0.656	0.701	0.868	**1**
Random Forest	0.789	0.880	0.816	0.801	0.886	0.813	**0.813**	**0.896**	**0.817**	**0.813**	**0.897**	**0.813**	**3, 4**
Gaussian Process	**0.813**	**0** **.897**	**0** **.813**	0.807	0.891	0.820	0.807	0.891	0.820	0.747	0.850	0.817	**1**

ACC = Accuracy, P = Precision. Bold: Better results

**Table 7:** Final result of the study.

CLASSIFIERS	DataSET 1—First Event			DataSET 1—Second Event			DataSET 1—Third Event		
	ACC	F1	P	ACC	F1	P	ACC	F1	P
Decision Tree	0.831	0.900	0.864	0.831	0.900	0.864	0.825	0.895	0.867

## References

[1] Espinel-Bermúdez M.C., Sánchez-García S., García-Peña C., Trujillo X., Huerta-Viera M., Granados-García V., Arias-Merino E.D. (2018). Associated factors with sarcopenia among Mexican elderly: 2012 National Health and Nutrition Survey. Rev. Médica Del Inst. Mex. Del Seguro Soc..

[2] Van Ancum J.M., Scheerman K., Jonkman N.H., Smeenk H.E., Kruizinga R.C., Meskers C.G., Maier A.B. (2017). Change in muscle strength and muscle mass in older hospitalized patients: A systematic review and meta-analysis. Exp. Gerontol..

[3] Martone A.M., Bianchi L., Abete P., Bellelli G., Bo M., Cherubini A., Marzetti E. (2017). The incidence of sarcopenia among hospitalized older patients: Results from the Glisten study. J. Cachexia Sarcopenia Muscle.

[4] Dreder A. (2017). Machine Learning Based Approaches for Identifying Sarcopenia-Related Genomic Biomarkers in Ageing Males.

[5] Hamrioui S., de la Torre Díez I., Garcia-Zapirain B., Saleem K., Rodrigues J.J.P.C. (2017). A Systematic Review of Security Mechanisms for Big Data in Health and New Alternatives for Hospitals. Wirel. Commun. Mob. Comput..

[6] Michael B. (2015). Machine Learning in Python: Essential Techniques for Predictive Analysis.

[7] Alonso S.G., de la Torre Diez I., Zapirain B.G. (2019). Predictive, Personalized, Preventive and Participatory (4P) Medicine Applied to Telemedicine and eHealth in the Literature. J. Med. Syst..

[8] Mugueta-Aguinaga I., Garcia-Zapirain B. (2017). Is Technology Present in Frailty? Technology a Back-up Tool for Dealing with Frailty in the Elderly: A Systematic Review. Aging Dis..

[9] De la Torre I., Cosgaya H.M., García-Zapirain B., López-Coronado M. (2016). Big data in health: A literature review from the year 2005. J. Med. Syst..

[10] Lupianez-Villanueva F., Anastasiadou D., Codagnone C., Nuno-Solinis R., Garcia-Zapirain Soto M.B. (2018). Electronic Health Use in the European Union and the Effect of Multimorbidity: Cross-Sectional Survey. J. Med. Internet Res..

[11] Coplade B.C. Tijuana, Baja California: COPLADE; 2017; p. 10. http://www.copladebc.gob.mx/publicaciones/2017/Mensual/Tijuana%202017.pdf.

[12] Castro E.M.M. (2019). Bioestadística aplicada en investigación clínica: Conceptos básicos. Rev. Médica Clínica Las Condes..

[13] Steffl M., Bohannon R.W., Sontakova L., Tufano J.J., Shiells K., Holmerova I. (2017). Relationship between sarcopenia and physical activity in older people: A systematic review and meta-analysis. Clin. Interv. Aging.

[14] Liu P., Hao Q., Hai S., Wang H., Cao L., Dong B. (2017). Sarcopenia as a predictor of all-cause mortality among community-dwelling older people: A systematic review and meta-analysis. Maturitas.

[15] Bianchi L., Abete P., Bellelli G., Bo M., Cherubini A., Corica F., Rossi A.P. (2017). Prevalence and Clinical Correlates of Sarcopenia, Identified According to the EWGSOP Definition and Diagnostic Algorithm, in Hospitalized Older People: The GLISTEN Study. J. Gerontol. Ser. A.

[16] Polan D.F., Brady S.L., Kaufman R.A. (2016). Tissue segmentation of computed tomography images using a Random Forest algorithm: A feasibility study. Phys. Med. Biol..

[17] Cassia Braga R. (2014). SciELO-Public Health-Name segmentation using hidden Markov models and its application in record linkage Name segmentation using hidden Markov models and its application in record linkage. Cad. De Saude Publica.

[18] Hodinka L. (2018). Sarcopenia, Frailty and Dismobility. Biomed. J. Sci. Tech. Res..

[19] Walston J.D. (2012). Sarcopenia in older adults. Curr. Opin. Rheumatol..

[20] Burns J.E., Yao J., Chalhoub D., Chen J.J., Summers R.M. (2020). A Machine Learning Algorithm to Estimate Sarcopenia on Abdominal CT. Acad. Radiol..

[21] Lenchik L., Boutin R.D. (2018). Sarcopenia: Beyond Muscle Atrophy and into the New Frontiers of Opportunistic Imaging, Precision Medicine, and Machine Learning. Semin. Musculoskelet Radiol..

[22] Qian D. (2020). Fully-automated Segmentation of Muscle Measurement on CT in Detecting Central Sarcopenia: A Trend of Standardization. Acad. Radiol..

[23] Barnard R., Tan J., Roller B., Chiles C., Weaver A.A., Boutin R.D., Lenchik L. (2019). Machine Learning for Automatic Paraspinous Muscle Area and Attenuation Measures on Low-Dose Chest CT Scans. Acad. Radiol..

[24] Graffy P.M., Liu J., Pickhardt P.J., Burns J.E., Yao J., Summers R.M. (2019). Deep learning-based muscle segmentation and quantification at abdominal CT: Application to a longitudinal adult screening cohort for sarcopenia assessment. Br. J. Radiol..

[25] Cernea A., Fernández-Martínez J.L., de Andrés-Galiana E.J., Fernández-Muñiz Z., Bermejo-Millo J.C., González-Blanco L., Caballero B. (2019). Prognostic networks for unraveling the biological mechanisms of Sarcopenia. Mech. Ageing Dev..

[26] Kang Y.-J., Yoo J.-I., Ha Y.-C. (2019). Sarcopenia feature selection and risk prediction using machine learning: A cross-sectional study. Medicine.

[27] Cui M., Gang X., Gao F., Wang G., Xiao X., Li Z., Wang G. (2020). Risk assessment of sarcopenia in patients with type 2 diabetes mellitus using data mining methods. Front. Endocrinol..

